# Evaluation of Nonradiative Clinical Imaging Techniques for the Longitudinal Assessment of Tumour Growth in Murine CT26 Colon Carcinoma

**DOI:** 10.1155/2013/983534

**Published:** 2013-07-02

**Authors:** Johanne Seguin, Bich-Thuy Doan, Heldmuth Latorre Ossa, Lauriane Jugé, Jean-Luc Gennisson, Mickaël Tanter, Daniel Scherman, Guy G. Chabot, Nathalie Mignet

**Affiliations:** ^1^Chemical, Genetic and Imaging Pharmacology Laboratory, Faculty of Pharmacy, Chimie ParisTech, Paris Descartes University, Sorbonne Paris Cité, INSERM U1022, CNRS UMR8151, 4 Avenue de l'Observatoire, 75006 Paris, France; ^2^Institut Langevin “Ondes et Images”, ESPCI ParisTech, CNRS UMR7587, INSERM U979, 1 Rue Jussieu, 75238 Paris Cedex 05, France

## Abstract

*Background and Objectives*. To determine the most appropriate technique for tumour followup in experimental therapeutics, we compared ultrasound (US) and magnetic resonance imaging (MRI) to characterize ectopic and orthotopic colon carcinoma models. *Methods*. CT26 tumours were implanted subcutaneously (s.c.) in Balb/c mice for the ectopic model or into the caecum for the orthotopic model. Tumours were evaluated by histology, spectrofluorescence, MRI, and US. *Results*. Histology of CT26 tumour showed homogeneously dispersed cancer cells and blood vessels. The visualization of the vascular network using labelled albumin showed that CT26 tumours were highly vascularized and disorganized. MRI allowed high-resolution and accurate 3D tumour measurements and provided additional anatomical and functional information. Noninvasive US imaging allowed good delineation of tumours despite an hypoechogenic signal. Monitoring of tumour growth with US could be accomplished as early as 5 days after implantation with a shorter acquisition time (<5 min) compared to MRI. *Conclusion*. MRI and US afforded excellent noninvasive imaging techniques to accurately follow tumour growth of ectopic and orthotopic CT26 tumours. These two techniques can be appropriately used for tumour treatment followup, with a preference for US imaging, due to its short acquisition time and simplicity of use.

## 1. Introduction

Colorectal cancer is the third leading cancer in the world in men and second in women with 1.2 million new cases identified in 2008. In terms of mortality, this cancer takes the second place after lung cancer with 608 700 deaths in 2008 [1]. It is therefore important to develop adapted models that could better represent the human pathology, in order to improve diagnostic methods or potential new therapies aimed at reducing the mortality rate of colon cancer. 

Murine colon tumour models can be obtained via *in situ* generation or via implantation of tumour fragments into appropriate syngeneic or immunosuppressed hosts. *In situ* models, including chemically induced, transgenic and spontaneous models, are considered less adapted for the evaluation of experimental therapies because of their inherent variability and the relatively long-time delay required for tumour growth and response to treatment [2]. While ectopic models are frequently employed owing to the ease of their implantation, orthotopic models are considered to better reflect the tumour physiological environment. In particular, significant differences between these models were found mainly in the level of growth factors and nutrients available and in their profile of tumour angiogenesis and metastasis [3]. Moreover, orthotopic tumours can metastasize into organs where they are usually found in spontaneously arising tumours [4–8].

In the present study, we have chosen to develop a murine orthotopic colon carcinoma model that could better reflect the clinical colon carcinoma situation, in order to be able to evaluate new antiangiogenic, antivascular, and antimetastatic approaches. To do so, we selected the colon carcinoma 26 (CT26) model because we have recently observed that this tumour is well vascularized and expresses cellular adhesion molecules, for example, alpha v beta 3 (*α*v*β*_3_) and E-selectin, which are important targets for antiangiogenic therapy [9]. 

Noninvasive imaging techniques applied to small animals have improved significantly in the past years. Magnetic resonance imaging (MRI), computed tomography (CT), positron emission tomography (PET), single-photon emission-computed-tomography (SPECT), ultrasound imaging (US), and bioluminescence imaging (BLI) are now being frequently used, and their performance is continuously improving [10, 11].

Although these various imaging techniques have been used for *in vivo* studies on small animals [10–12], the information available is scarce concerning noninvasive growth evaluation of colon tumours growing orthotopically. In this study, we therefore focused on the evaluation of quantitative tumour growth parameters available with nonradiative and noninvasive imaging techniques on the two CT26 tumour models. The advantages and limitations of each imaging technique for the two models are presented.

## 2. Materials and Methods

### 2.1. Cells

The CT26 cell line was originally obtained from an undifferentiated colon carcinoma chemically induced by *N*-nitroso-*N*-methylurethan [13] that was later cloned to obtain the stable CT26 cell line [14]. This cell line was purchased from American Type Culture Collection (ATCC, CRL-2638, LGC Standards, Molsheim, France) and cultured at 37°C in a 5% CO_2_-humidified atmosphere in Dulbecco's Modified Eagle Medium (DMEM, Gibco) containing 10% fetal bovine serum (FBS, Gibco Life technologies), 100 *μ*M of streptomycin, and 100 U/mL of penicillin. 

### 2.2. Animals

Studies were carried out in Balb/c female mice (Janvier, St Genest de Lisle, France), aged from 6 to 7 weeks. Animal experiments were conducted according to European and national guidelines and were approved by the institutional ethics committee.

### 2.3. Ectopic Tumour Implantation

A mouse bearing a subcutaneous CT26 tumour was sacrificed, the tumour was resected, placed into DMEM culture medium, and cut into fragments of 20–30 mm^3^. These fragments were evaluated to contain approximately 9 × 10^5^ tumour cells, using hemocytometer measurement after trypsin disaggregation. The tumour fragments were transferred into sterile phosphate buffer saline and inserted subcutaneously using a 12 gauge trocar (38 mm) into the mouse flank previously disinfected with alcohol. For the CT26 line, tumours were implanted every two weeks. In these conditions, the take rate was 100%. 

### 2.4. Surgical Orthotopic Implantation

Allografts were prepared from ectopic tumours passaged as described previously in Section 2.3, and tumours were implanted orthotopically as described in [15, 16]. Briefly, mice were anesthetized and their abdomen was shaved and disinfected with Betadine (Meda Pharma, France). A laparotomy was conducted in order to have access to the abdominal cavity and the tumour fragment was sutured onto the caecum, after the removal of the serosa, to allow a good diffusion of the tumour cells into the colon mucosa, as described in [16, 17]. Finally the muscular tissue and the skin were stitched.

### 2.5. *Ex Vivo* Characterization

In order to evaluate histology and tumour weight evolution of both models, five mice per model and per time point were implanted. At the indicated time points (5, 10, 15, or 21 days), mice were sacrificed and their tumours resected. A photograph of each tumour was captured using a Canon Digital IXUS 80 IS camera. Finally, the tumour was weighed and flash frozen in liquid nitrogen. Ten-micron frozen tissue sections were obtained in the middle of the tumour and stained with haematoxylin. The orthotopic tumours were compared with a caecum of healthy mice and the ectopic CT26 tumour.

### 2.6. Immunohistochemistry

Ten-micron frozen tissue sections were placed on Polysine slides (Polysine, CML, Nemours, France). Immunostaining for the evaluation of the vascularity was performed using a three-step procedure as previously described in [9, 18]. Briefly, the tissue sections were first incubated with a rat anti-mouse CD31 (PECAM-1) monoclonal antibody (BD Biosciences, le Pont de Claix, France; 15.6 *μ*g/mL) for 2 h at 37°C. The sections were next incubated for 30 min with goat anti-rat biotinylated antibody (0.5 mg/mL) and finally with the streptavidin-conjugated peroxidase (Sigma, 1/400 dilution). The staining was carried out with 3,3′-diaminobenzidine (DAB, Sigma) substrate to obtain a brown precipitate. Tissues were counterstained with Gill's haematoxylin to visualize cell nuclei. 

For each tumour, immunohistochemistry assessment was obtained in the central slice of the tumour. In this slice, ten representative images were realized and analysed. All stains were performed at the same time and images digitization was made under identical lighting conditions to insure that analyses from different slides were comparable. 

### 2.7. Assessment of Perfusion with Hoechst 33342

In order to assess *ex vivo* tumour perfusion, mice were injected i.v. with 100 *μ*L of Hoechst 33342 solution (10 mg/kg dissolved in saline, Molecular Probes, Eugene, OR, USA) 1 min before sacrifice. Tumours were removed and flash frozen in liquid nitrogen. Ten-micron frozen tissue sections were realized in the middle of the tumour and were placed on Polysine slides. Tumour section was incubated 2 h at 37°C with the rat anti-mouse CD31 monoclonal antibody (BD Biosciences, le Pont de Claix, France; 15.6 *μ*g/mL) and further incubated with an anti-rat fluorescein isothiocyanate (FITC) secondary antibody (Sigma) at 1/400 dilution for 45 min in the dark. Slices were mounted in aqueous media (Immu-mount, Thermo Fisher Scientific, France) and observed under fluorescence microscopy (Axiophot, Zeiss, Wetzlar, Germany) equipped with a camera Retiga 2000 (Q Imaging, Surrey, Canada). Photographs were realized in the edge (*n* = 4) and in the centre (*n* = 4) of the tumour. Intensity of fluorescence emitted by the perfused cells was quantified with ImageJ.

### 2.8. Immunostaining Quantitative Analysis

For the characterization of the tumour vascularization, we developed a method for the quantification of various markers using the ImageJ software [19]. The images were first segmented to isolate the positive structure of interest and the images were then transformed into binary ones. Finally, the edges of the stained cells were selected and the various parameters, such as counts and areas, were determined. Microvascular density (MVD) corresponds to the mean of the number of vessels per mm^2^, per tumour, and per time point. 

### 2.9. 3D Vasculature Visualization by Fluorescence Imaging

Mice were anaesthetized with a mixture of ketamine/xylazine (80 and 10 mg/kg, resp.), and a solution of albumin alexa fluor 594 (Molecular probes, Life Technologies SAS, Saint Aubin, France) was injected intravenously (i.v.) into the tail vein (0.5 mg/mL, 100 *μ*L per mouse). A subcutis incision near the tumour for the ectopic model and a laparotomy for orthotopic model were realized in order to visualize the tumour vascularization under a Leica fluoromacroscope (Z6 APO), and the image acquisition was performed with a Leica camera (DFC 340Fx). In our experimental conditions, it was possible to visualize the tumour vascularisation in the depth of 3.1 mm to 0.09 mm.

### 2.10. Magnetic Resonance Imaging (MRI)

The MRI studies were performed using an MRI spectrometer 7T PharmaScan (Bruker, France, ESPCI Imaging Platform) dedicated to small animal experimentation. Images were recorded using a 36 mm inner diameter birdcage Bruker RF probe. Mice were anaesthetized using 1.5% isoflurane gaz with a mixture of air/O_2_ (1 L/min : 0.5 L/min). Physiological parameters of temperature and breathing rates were controlled.

#### 2.10.1. Assessment of Tumour Volume

In order to determine the tumour volume, Gradient Echo imaging sequence was performed. Thirty axial slices of 0.83 mm thickness with a plane resolution of 0.208 mm were acquired during 3 min, which cover the entire volume of the tumour. For the calculation of the tumour volume, the tumour region was delineated on each section with ImageJ software [19]. The area of each region of interest (in mm^2^) was multiplied by the thickness of the slices (0.83 mm) to calculate the volume (in mm^3^) of each slice. To obtain the volume of the tumour, the volume of each slice was summed.

#### 2.10.2. Assessment of Haemorrhagic Necrosis

Five axial *T*_2_-weighted images of the centre of tumour were obtained thereafter for an accurate depiction of the anatomy and the quantification of necrosis by using a Spin Echo sequence with an echo time TE of 54 ms. These slices have a thickness of 0.6 mm and a plane resolution of 0.123 mm for an acquisition time of 15 min. Hemorrhagic necrosis containing deoxygenated haemoglobin appears mainly in hyposignal. For the calculation of the hyposignal area a threshold of 8% was applied in the images from *T*_2_-weighted images. The percentage of hyposignal area was obtained by the mean of the number of pixels under the threshold per slice divided by the slice area.

#### 2.10.3. Assessment of Oedemas

*T*_2_-weighted Spin Echo images were recorded to assess inflammatory oedemas [20]. *T*_2_ maps were computed from these multicho SE images by using the following equation (Paravision software, Bruker):

(1) $$\begin{array}{c} S=S_{0}\exp\left(-\frac{\text{TE}}{T_{2}}\right), \end{array}$$

where *S* is the signal intensity obtained at each echo time (TE) and *S*_0_ is the magnitude of the signal without relaxation. 

#### 2.10.4. Dynamic Contrast Enhanced (DCE) MRI for the Assessment of Tumour Blood Perfusion

Multislice relaxation rate (*R*_1_ = 1/*T*_1_) maps were obtained by using saturation recovery gradient echo images with variable flip angles before and after contrast agent administration [21]. After baseline acquisitions, DOTAREM was administrated i.v. at a dose of 0.4 mmol/kg, and postcontrast DCE images were acquired over approximately 15 min. Axial images were collected from at least two to three slices through the whole tumour. Muscles were sampled to estimate the concentration of contrast agent in the blood. Images processing, region of interest (ROI), selection and MR data analyses were carried out using Paravision 5 and ImageJ. The *R*_1_ and maximal signal intensity (*S*_max⁡_) were calculated according to Seshadri et al. [22]. The Delta *R*_1_ of the tumour (Δ*R*_1*t*_) and the muscle (Δ*R*_1*m*_) were performed to calculate the slope and *y*-intercept value at time zero. The slope represents the permeability of tumour vessels to DOTAREM, and the *y*-intercept provides a measure of tumour vascular volume [21, 23].

### 2.11. Ultrasound (US) Imaging

Ultrasound imaging was performed using an ultrafast ultrasound device (Aixplorer, Supersonic Imagine, Aix en Provence, France) coupled with a high-frequency ultrasound probe (central frequency 15 MHz, 256 elements, Vermon, Tours, France). The system can reach a frame rate of 20 000 images/s with special resolution of 100 *μ*m. Mouse anaesthesia was performed using isoflurane, in the same conditions as described above for MRI imaging. 

#### 2.11.1. Assessment of Tumour Volume

By using classical ultrasound imaging, the so-called B-mode, the operator localized the tumour within the imaging plane in real time by placing the probe on the anaesthetized mouse. Once the probe was positioned, B-mode acquisitions were performed to obtain a morphological image of the tumour in two perpendicular imaging planes (axial and sagittal) in order to measure their largest diameters. Thus, three diameters (two in the axial and one in the sagittal planes) were recorded, giving access to volume quantification according to the following spheroid formula: (*π*/6) × *L* × *W* × *H*, where *H* is the height, *L* is the length, and *W* is the width in cm [24]. 

#### 2.11.2. Assessment of Blood Flow

To observe the vascular network, colour Doppler measurements were performed with the same probe at 15 MHz. The blood velocity was color coded in scales ranging from 0 to 2 m/s.

### 2.12. Statistical Analysis

 Analysis was performed with GraphPad Prism software. In order to determine statistical difference between 2 groups of mice, the Mann-Whitney *U* test was used with a *P* value <0.05 considered as significant. 

## 3. Results

### 3.1. *Ex Vivo* Characterization of Ectopic and Orthotopic Carcinoma Mouse Models

**Figure 1:**
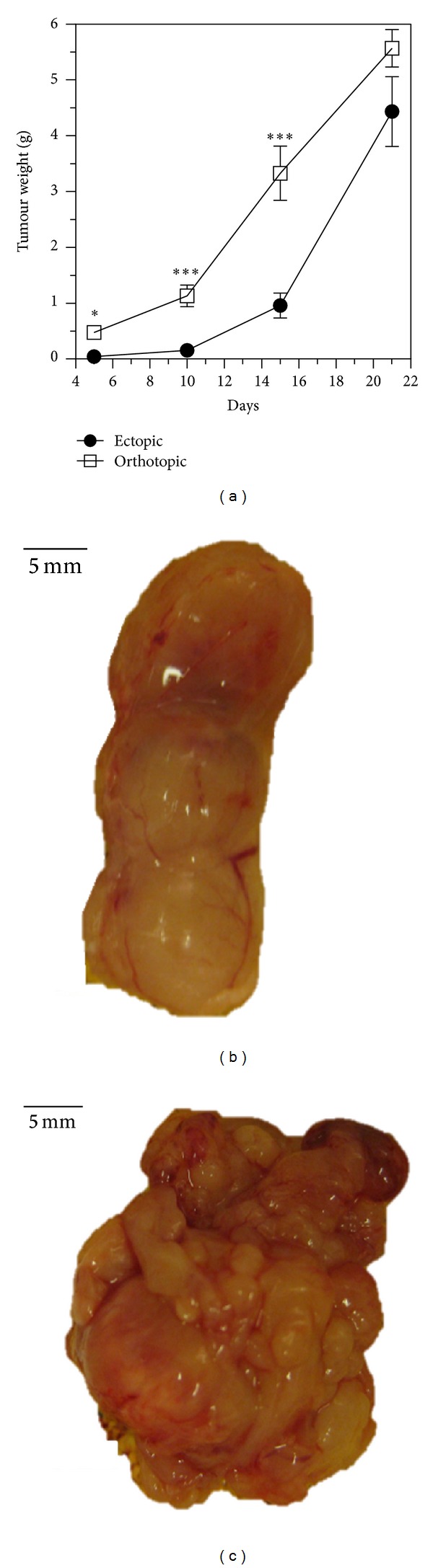
*Ex vivo* characterization of ectopic and orthotopic colon carcinoma CT26 implanted in mice. (a) Tumours were harvested from Balb/c mice at the indicated days and weighed. The means ± SEM of 5 tumours are presented. The asterisks indicate a significant difference between the orthotopic (squares) and the ectopic group (circles) using the Mann Whitney test. Photographs of a representative tumour on day 21 are shown in (b) for the ectopic implantation and in (c) for the orthotopic tumour (scale bar, 5 mm).

We first characterized the ectopic and orthotopic colon carcinoma CT26 models using invasive methods. To do so, tumours were resected and weighed at different times.  Figure 1(a) depicts the CT26 tumour weight evolution for the ectopic and the orthotopic implantation during 21 days. Representative tumour photographs taken on day 21 are shown in Figure 1(b) for the ectopic (subcutaneous) and in Figure 1(c) for the orthotopic (caecum) implantation.

**Figure 2:**
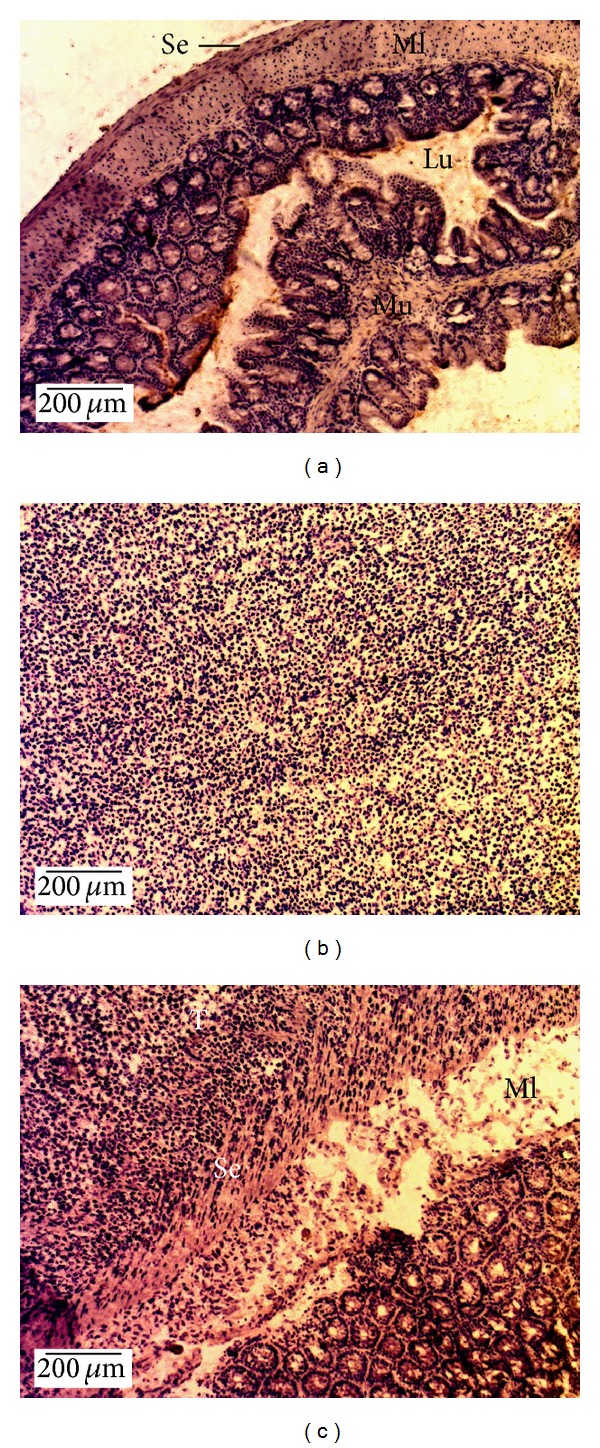
Histology of CT26 tumours implanted into the intestinal mucosa. Histological images were obtained using haematoxylin counterstain for healthy caecum without tumour (a), ectopically implanted CT26 tumours (b), and orthotopic CT26 tumour (c). Tumours were harvested 21 days after tumour implantation. Original magnification 100x, scale bar 200 *μ*m). Lu, lumen; Ml, muscularis; Mu, mucosa; Se, serosa; T, tumour.

We next verified the correct implantation of the orthotopic tumour into the intestinal mucosa.  Figure 2 presents a healthy caecum without tumour (Figure 2(a)) and an ectopically implanted CT26 tumour (Figure 2(b)) where the tumour cells can be seen. In Figure 2(c), the histological examination of orthotopic slices clearly shows the invasion of the caecal serosa and the muscularis by the tumour cells.

### 3.2. Histological Characterization of Cellularity and Vessel Density

**Figure 3:**
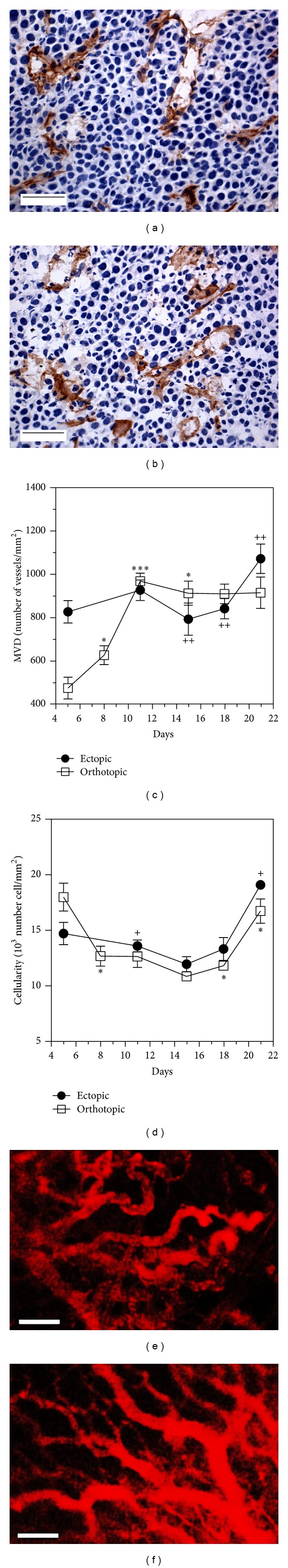
Histological characterization of cellularity and vessel density of CT26 tumours. Histological images obtained after immunohistochemistry of PECAM 1 (positive staining in brown) and haematoxylin counterstain for ectopic (a) and orthotopic (b) 15 days after tumour implantation (original magnification 400x, scale bar 50 *μ*m). Microvascular density (MVD) quantification (c) and cellularity (d) are presented as a function of time (mean ± SEM). The 3D vascular organization was investigated, 15 days after tumour implantation, by fluorescence using Alexa fluor 594-labeled albumin injected i.v. (e) Ectopic, (f) orthotopic, scale bar, 100 *μ*m.

Cellularity and vessel density of the colon tumours were determined at different times by histological analyses using PECAM-1 and haematoxylin to stain the tumour cells. As depicted in Figure 3(a) for the ectopic tumour and Figure 3(b) for the orthotopic tumour, the sections were relatively similar in both implantation sites, with homogenously dispersed cancer cells and vessels.

Quantitative analyses of the histological sections allowed for the determination of tumour microvascular density (MVD) and cellularity.  Figure 3(c) presents the MVD for both models. For the ectopic tumour, the MVD was found relatively stable during 21 days ranging from 800 to 1100 vessels/mm². The orthotopic model CT26 tumours presented a significantly lower MVD level in the early days after tumour implantation compared to the ectopic ones (476 ± 50 vessels/mm² on day 5) but increased rapidly thereafter to reach the ectopic MVD values on day 11 (968 ± 36 vessels/mm²). 

Cellularity presented in Figure 3(d) decreased significantly for both models between the 5th and the 11th days. Cellularity values decreased from 14.7 ± 1.0 × 10^3^ to 13.6 ± 0.5 × 10^3^ cells/mm^2^ for the ectopic model and from 18.0 ± 1.3 × 10^3^ to 12.6 ± 1.0 × 10^3^ cells/mm^2^ for the orthotopic model, respectively. After the 11th day until the 18th day the cellularity remained stable for both models. A significant increase in cellularity was however observed for both tumour implantation sites after day 18.

### 3.3. 3D Vascular Organization of Ectopic and Orthotopic Tumours

The vascular organization was observed on both models 15 days after tumour implantation using a 3D evaluation method based on spectrofluorescence. Figures 3(e) and 3(f) present the vascular network of CT26 colorectal tumours obtained after the intravenous injection of labelled albumin 24 h after tumour implantation. It can be observed that both CT26 tumours were highly vascularized and presented vessel disorganization with several shunts (Figures 3(e) and 3(f)). 

### 3.4. Longitudinal Growth Monitoring of CT26 Tumours by MRI

**Figure 4:**
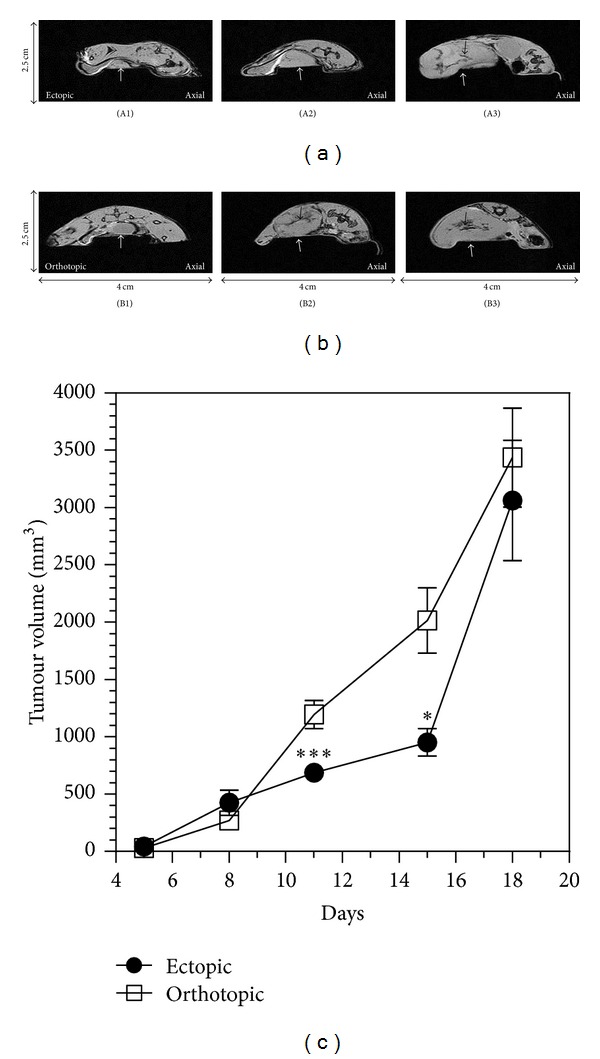
*T*_2_* weighted images showing the ectopic and orthotopic CT26 tumour. *T*_2_* weighted imaging provides anatomical axial images of ectopic (a) and orthotopic model (b) at days 5, 11, and 15. White arrows indicate the tumour localisation. Black arrows show the hyposignal regions. (c) Evaluation of tumour volume with images quantification for ectopic and orthotopic model as a function of time.

We next explored the characterization of colon carcinoma CT26 models using MRI. Coronal image was obtained from *T*_2_*-weighted sequences in which axial slices were positioned in the tumour area (see Figure 1 in the supplementary material available online at http://dx.doi.org/10.1155/2013/983534). In these axial slices, tumour burden was easily detected without the use of a contrast agent. The tumour mass appeared in grey and the outline was relatively well defined as shown in Figures 4(a) and 4(b) (white arrows). From these images, it was possible to calculate the tumour volume by the addition of the surface areas of the consecutive axial slices taken in the tumour mass. Small tumours, ranging from 15 to 20 mm^3^ measured at day 5, to large tumours up to 3000–3400 mm^3^ measured at day 18, are shown on Figure 4(c). Tumour growth obtained by MRI followed a similar profile for both models with a significant difference on days 11 and 15. 

*T*_2_ weighted images provide valuable information on extracellular water such as inflammatory oedemas displaying hypersignal [20] and hemorrhagic necrosis containing deoxygenated haemoglobin that appears mainly in hyposignal [25, 26]. 

In a first place, we measured the global mean *T*_2_ value in ectopic and orthotopic tumours (Table 1). For the ectopic model, *T*_2_ decreased significantly (*P* < 0.05) from day 5 (56.4 ± 3.0 ms) to day 11 (46.1 ± 1.5 ms) and remained stable beyond this time point (48.5 ± 1.0 and 47.5 ± 1.5 ms for days 15 and 18, resp.). For the orthotopic model a similar profile was observed. The absence of hypersignal indicated that there was no detectable inflammatory oedema from day 11 to day 18 [25, 26]. The slightly higher *T*_2_ value on day 5 could indicate a slight inflammation due to tumour implantation.

In a second place, the percentage of hyposignal was calculated. The fraction of hyposignal (Figure 4(A3), 4(B2), and 4(B3), black arrows) in the tumour mass could be measured and is expressed as percentages in Table 2. Late stage tumours (from day 11 to day 18) presented a sharp increase in the hyposignal area in the ectopic model (6.6 ± 0.8% to 22.5 ± 2.9%), whereas it remained relatively stable in the orthotopic one (14.0 ± 3.6% to 12.7 ± 2.7%).

**Figure 5:**
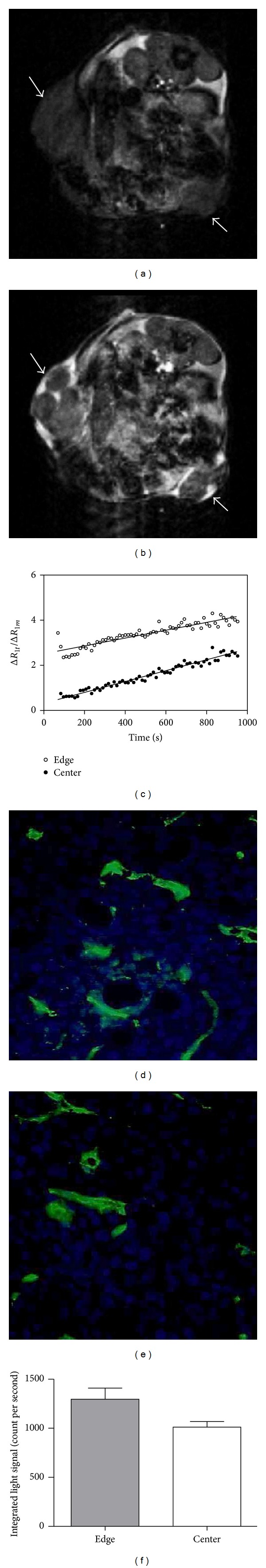
Assessment of perfusion and permeability data *in vivo* by MRI and *ex vivo* by immunofluorescence. (a) MRI image of CT26 tumour (white arrow) before Gd contrast agent injection and (b) MRI image of CT26 tumour (white arrow) after Gd contrast agent injection. (c) Representation of Δ*R*_1*t*_/Δ*R*_1*m*_ as a function of time after injection of Gd contrast agent in the edge and the centre of ectopic CT26 tumours. *Ex vivo* immunofluorescence in the edge (d) and in the centre (e) of ectopic CT26 tumour showing endothelial cells (CD31-FITC, green labeling) and perfused tumour cells (Hoechst 33342, blue labeling). (f) Intensity of perfused tumour cells in the edge and in the centre of ectopic tumour cells quantified by ImageJ.

The DCE MRI experiments provided quantitative data on the tumour perfusion changes (Figures 5(a)–5(c)).  Figure 5 depicts the tumour before (Figure 5(a)) and after (Figure 5(b)) injection of DOTAREM. The ratio of the Δ*R*_1_ in the tumour (Δ*R*_1*t*_) and the Δ*R*_1_ of the muscle (Δ*R*_1*m*_) are represented in Figure 5(c). We found that the Δ*R*_1*t*_/Δ*R*_1*m*_ ratio on the edge of the tumour was higher than the Δ*R*_1*t*_/Δ*R*_1*m*_ ratio calculated in the centre of the tumour (Figure 5(c)), showing a higher tumour vascular volume in the peripheral regions as compared to the centre areas. The lower Δ*R*_1*t*_/Δ*R*_1*m*_ obtained in the centre might also derive from a weaker accumulation of the contrast agent in the centre of the tumour due to a different tumour environment. The fact that the Δ*R*_1*t*_/Δ*R*_1*m*_ (centre) slope was slightly steeper as compared to the Δ*R*_1*t*_/Δ*R*_1*m*_ (edge) could also suggest a higher vascular permeability in the centre of the tumour.

Moreover, the intravenous injection of a chromophor compound 1 min before sacrifice provided convergent histological data about tumour perfusion (Figures 5(d) and 5(e)). If we examine tumour perfusion picture taken in the edge of the tumour (Figure 5(d)), the staining of the nucleus (blue) was slightly higher than in the pictures taken in the centre of the tumour (Figure 5(e)). This confirmed a higher perfusion of the cells in the edge of the tumour (Figure 5(f)), as also evidenced by CD31 immunofluorescent staining. 

Overall, *ex vivo* fluorescent data and *in vivo* MRI data are consistent with a lower perfusion rate in the centre of the tumour.

### 3.5. Longitudinal Growth Monitoring of CT26 Tumours by Ultrasound (US) Imaging

**Figure 6:**
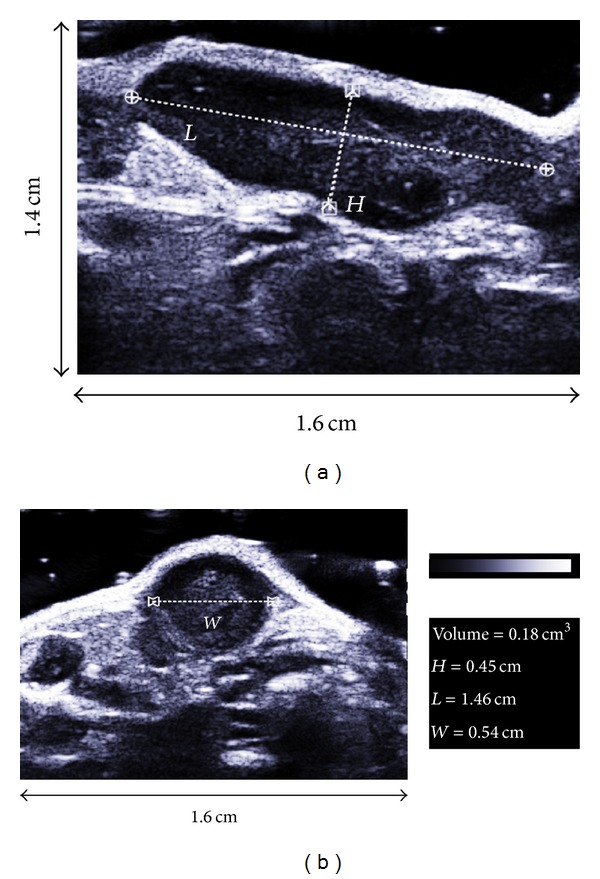
Method used for the calculation of tumour volume by ultrasound imaging. Images show an example from an ectopic tumour on day 15 using a saggital (a) and an axial (b) view.

We also evaluated echography as a noninvasive US imaging technique and assessed its usefulness for the monitoring of colon CT26 growth in mice. As presented in Figure 6, the B-mode images showed a contrast difference due to a hyperechogenicity of the skin and a hypoechogenicity of the tumour. The resulting contrast enhancement allowed to delineate the tumour margins and measure the tumour height, length, and width, which allowed the calculation of tumour volumes. Examples of such measurements on a representative ectopic tumour on day 15 are presented in Figure 6 depicting two perpendicular views ((a) and (b)). These images allowed to quickly measure the three dimensions necessary to assess the tumour volume as stated in Section 2.

**Figure 7:**
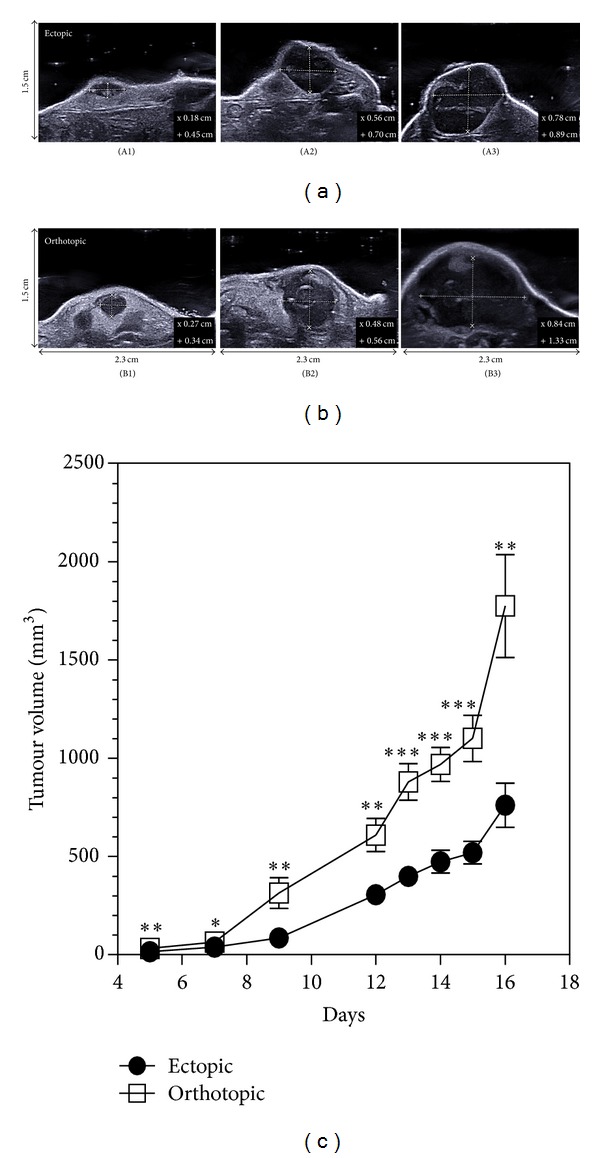
Ultrasound longitudinal monitoring of tumour volume. Representative images used for the quantification of tumour volume for ectopic (*n* = 13) on days 5 (A1), 12 (A2), and 15 (A3) and orthotopic (*n* = 13) on days 5 (B1), 12 (B2), and 15 (B3).

Figure 7 depicts representative tumour images from both implantation sites on days 5, 12, and 15. Using US, tumour measurement could be obtained as early as 5 days after tumour implantation for both models and allowed an easy non invasive monitoring of tumour growth. US imaging allowed the measurement of small tumours on day 5 of 15.8 ± 2.1 mm^3^ for the ectopic site (Figure 7(A1)) and 31.9 ± 4.0 mm^3^ for the orthotopic one (Figure 7(B1)). On day 15, the tumour volume values reached 519.7 ± 56.6 mm^3^ (Figure 7(A3)) and 1101.0 ± 117.0 mm^3^ (Figure 7(B3)) for the ectopic and orthotopic models, respectively.

**Figure 8:**
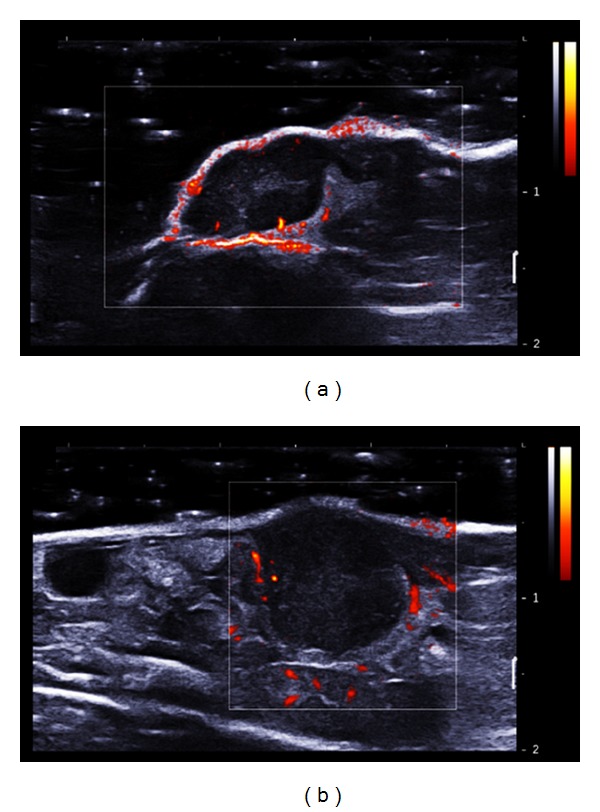
Visualization of tumour vascularization with Doppler mode (standard Doppler fixed between 0 and 2 m/s). (a) CT26 ectopic tumour and (b) orthotopic CT26 tumour 15 days after tumour implantation. The blood velocity was color coded in scales ranging from 0 to 2 m/s.

At last, blood flow could be obtained thanks to the Doppler mode. As observed in Figure 8, only large blood vessels could be detected in CT26 models due to the sensitivity of the system. 

## 4. Discussion

In this study we have evaluated two non invasive clinical imaging modalities to provide information on mice implanted ectopically and orthotopically with CT26 colon carcinoma. Even though the CT26 orthotopic model has previously been described [27, 28], the simultaneous evaluation and comparison of methods including histology, spectrofluorescence, MRI, and echography have not been reported before. We therefore compared these non invasive imaging modalities which could advantageously be applied in the followup of preclinical experimental therapies aimed at finding better treatments for human colorectal cancer. 

Examination of the histological results showed that the MVD value was significantly lower for the orthotopic model in the early time points as compared to the ectopic implantation. These values are of importance considering that the angiogenic phase is delayed in the orthotopic model, as compared to the ectopic model. This indicates that the orthotopic implantation would be a more appropriate model for the evaluation of antiangiogenic experimental therapy. With regard to antivascular approaches, both models appear appropriate after day 11, where the MVD was almost equivalent. 

Our data using 3D fluorescence imaging with labelled albumin indicate that antivascular approaches could be followed using this technique because it gives valuable data on vessel density and vessel shunts. However, it should be noted that vessel imaging using labelled albumin only allows visualizing the superficial vascular system and that quantification still needs to be addressed.

MRI provided highly resolved anatomical images and enabled to monitor the tumour growth of both CT26 models from early stage (day 5) to late growth stage (day 21). The anatomical resolution allowed the precise determination of 3D tumour volume in both models. This volume was correlated to *ex vivo* measurements as also previously shown on adrenal tumours [29]. Moreover, MRI anatomic and functional data, such as oedematous lesions, haemorrhagic regions, blood flow, and perfusion, could be helpful in tumour diagnosis and staging [30]. In addition, the *T*_2_ parameter gives valuable information on haemorrhagic necrotic area and oedema [31]. However, the *T*_2_ parameter needs to be chosen precisely to allow the detection of differences in tumour homogeneity. Indeed, the definition of percent of hyposignal with *T*_2_-weighted image was found to be more representative of tumour heterogeneity compared to global assessment of *T*_2_ values.

The DCE MRI experiments provided to quantify perfusion changes in tumour. The results obtained in this study are in good agreement with histological results previously reported [32]. The higher signal intensity obtained by DCE-MRI in the edge of the tumour using a contrast agent was consistent with a more important labelling of perfused tumour cells in the tumour periphery assessed by *ex vivo* fluorescent study. Therefore, perfusion experiments could be relevant to assess antivascular activity *in vivo* of vascular disrupting agents. Indeed, the effect of antivascular disrupting agents will affect the haemorrhagic zone often located in the centre of tumours and induce the formation of a viable rim which could then be measurable in a noninvasive way. 

Moreover, thanks to magnetic resonance elastography (MRE), the viscoelastic properties of the tissue can be obtained to better characterise the tumour [33, 34]. In previous experiments, we have shown that increased elasticity is positively correlated with increased vascularisation for both ectopic and orthotopic CT26 carcinoma mouse models [35]. This technique could be helpful to monitor changes in tissue elasticity during treatment, particularly with antivascular drugs [35].

As described above, MRI presents several advantages and is particularly adapted to follow tumour growth and tumour stage during treatment. However, three critical points should be considered: MRI remains an expensive technique, the operator should be qualified, and this modality could take from 5 to 20 min acquisition time per mouse (Table 3).

Concerning the results obtained using ultrasound imaging, data provided a good estimation of tumour volume in both implantation models, as expected [24]. Moreover, progress of these techniques has been so rapid that US-3D imaging is now easily available. It might still require longer acquisition times, but it gives an excellent picture of the tumour burden as shown for CT26 ectopic tumours (Figure 2 in supplementary material).

Tumour blood flow could be assessed by ultrasound imaging without the use of contrast agent by using appropriate probes and Doppler mode. Unfortunately, in our study, we could not detect blood flow in the tumour centre with the 15 MHz probe used. This is probably due to the low sensitivity of the ultrasound device (2 m/s) that could not detect such small vessels. The lack of visualization of tumour microvessels is also probably due to a lower venous pressure which causes flow stasis [36]. It has been shown that, following an antivascular treatment, tumour blood flow reduction and revascularisation can be assessed using US imaging [37]. The probe used in the Goertz et al. study [37] had a frequency of 25 MHz, which allowed a sensibility of 0.75 mm/s which is more adapted to visualize tumour microvessels. Increased ultrasound sensibility could also be reached with sensitive Doppler. Such a tool could be more adapted for tumour vascularization monitoring [38]. 

Tumour elastic properties could also be measured with US imaging. As a matter of fact, shear wave elastography has also been developed and is now used in the clinic for breast tumour grade determination [39, 40]. We are presently evaluating this technique for the assessment of elastic properties of CT26 tumours, and its potential relevance in the follow up of antitumour therapies is being investigated. 

Finally, US imaging was found to be easy to perform and required only 1 to 5 min per mouse. This technique can advantageously be applied to the assessment of longitudinal CT26 tumour growth in the context of antitumour treatment (cf. Table 3).

## 5. Conclusions

In this study, non-invasive clinically available imaging techniques were evaluated to detect and follow up the growth of CT26 colon tumour. MRI and ultrasound techniques were shown to be particularly suitable for the accurate follow up of CT26 colon carcinoma growth in both ectopic and orthotopic mouse models. These imaging techniques and their pertinent parameters related to tumour growth, haemorrhagic areas, and perfusion could be assessed on CT26 mouse models. This first set of information is very important to define valuable imaging parameters that could be helpful for the diagnosis of tumour grade in the clinic and also for the monitoring of new antitumour approaches.

## Supplementary Material

The first figure in Supplementary Material: “Method used for the calculation of tumour volume by MRI. Images show an example from an ectopic tumour on day 15 using a coronal axe (A), and an axial view (B, in green).”
The second figure in Supplementary Material: “Ultrasound imaging of CT26 ectopic tumour. Axial plan (A), sagittal plan (B), coronal plan (C) and 3D view (D). Acquire with 8 MHz probe (192 elements, pitch of 0,2 mm, Aixplorer, Supersonic Imagine, Aix en Provence, France)”.

## Figures and Tables

**Table 1:** *T*_2_ values as a function of model type during longitudinal monitoring by MRI.

Days	Ectopic			Orthotopic		
	Mean (ms)	SD	*N*	Mean (ms)	SD	*N*
5	56.4	3.0	6	53.8	6.5	7
11	46.1	1.5	8	49.1	3.2	6
15	48.5	1.0	3	47.2	3.2	7
18	47.5	0.8	6	46.1	2.2	6

SD: standard deviation; *N*: number of mice.

**Table 2:** Percent of hyposignal in the tumour mass for CT26 ectopic and orthotopic models from day 8 to day 18.

Days	Ectopic			Orthotopic		
	Mean	SEM	*N*	Mean	SEM	*N*
8	ND			10.2	0.8	6
11	6.6	0.8	6	14.0	3.6	5
15	15.9	2.4	7	15.4	1.6	6
18	22.5	2.9	5	12.7	2.7	5

ND: not detected; SEM: standard error of the mean; *N*: number of mice.

**Table 3:** Comparison of MRI and US techniques to image mice bearing CT26 tumours.

Method	Spatial resolution (mm)	Smallest detectable tumour (volume mm^3^)	Analysis time (min)	Main advantages	Main disadvantages
MRI	0.123	14.47	15	(i) Real 3D volume (ii) Anatomic information (iii) Complementary data, functional information	(i) Expertise needed (ii) Duration of data analyses (iii) Operating cost

US	0.100	4.19	<5	(i) User friendly technique (ii) Volume can be measured directly	(i) Volume estimation (ii) Hairless mouse or shaved zone of observation needed (iii) Poor resolution
