# Supplementary material for: Evaluation of Nonradiative Clinical Imaging Techniques for the Longitudinal Assessment of Tumour Growth in Murine CT26 Colon Carcinoma
Source: Int J Mol Imaging. 2013 Jul 2;2013:983534. doi: 10.1155/2013/983534 (PMC3713650; doi:10.1155/2013/983534)
Supplement: Supplementary file 1 — The first figure in Supplementary Material: “Method used for the calculation of tumour volume by MRI. Images show an example from an ectopic tumour on day 15 using a coronal axe (A), and an axial view (B, in green).” The second figure in Supplementary Material: “Ultrasound imaging of CT26 ectopic tumour. Axial plan (A), sagittal plan (B), coronal plan (C) and 3D view (D). Acquire with 8 MHz probe (192 elements, pitch of 0,2 mm, Aixplorer, Supersonic Imagine, Aix en Provence, France)”. [file 983534.f1.pdf]

Supplementary materials

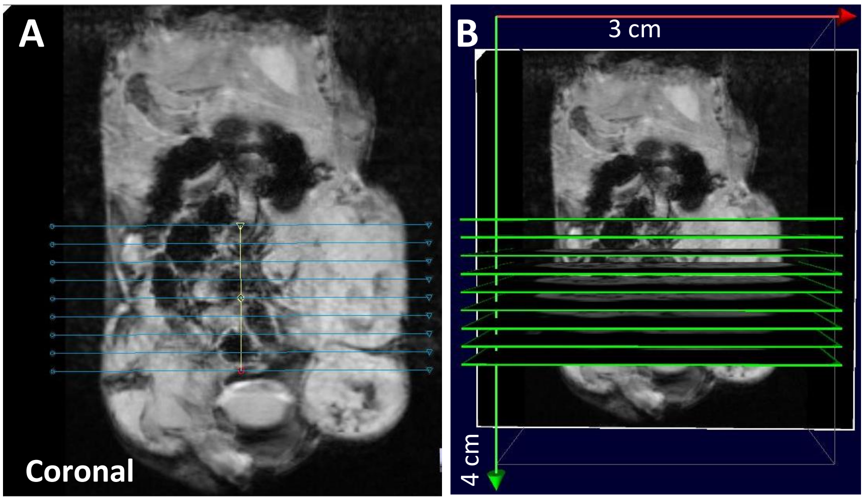

Figure 9: Method used for the calculation of tumour volume by MRI. Images show an example from an ectopic tumour on day 15 using a coronal axe (A), and an axial view (B, in green).

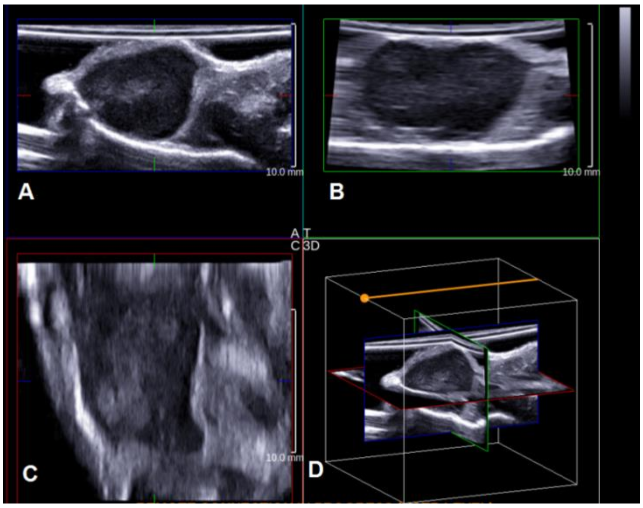

Figure 10: Ultrasound imaging of CT26 ectopic tumour. Axial plan (A), sagittal plan (B), coronal plan (C) and 3D view (D). Acquire with 8 MHz probe (192 elements, pitch of 0,2 mm, Aixplorer, Supersonic Imagine, Aix en Provence, France).
